# Comparative Evaluation of Boron Sorption Dynamics on Zeolites in Irrigation Waters: An Isothermal Modeling Approach

**DOI:** 10.3390/molecules29112545

**Published:** 2024-05-28

**Authors:** Dámaris Núñez-Gómez, Juan José Martínez-Nicolás, Pilar Legua, Carlos Giménez-Valero, Alejandro Andy Maciá-Vázquez, Pablo Melgarejo

**Affiliations:** Plant Production and Microbiology Department, Miguel Hernandez University (UMH), Ctra. Beniel Km 3.2, 03312 Orihuela, Alicante, Spain; dnunez@umh.es (D.N.-G.);

**Keywords:** boron sorption, zeolites, isothermal models, irrigation water, pollutant removal

## Abstract

Efficient boron removal from irrigation waters is crucial for sustainable agriculture, as elevated levels of boron can be toxic to many plants, limiting growth and crop productivity. In this context, the present study investigated the sorption equilibrium of boron using zeolites in two types of aqueous matrices: a synthetic solution containing only boron and natural irrigation waters. Through the application of various isothermal sorption models (Langmuir, Freundlich, Sips, Toth, Jovanovic, Temkin, Dubinin–Radushkevich, and Redlich–Peterson), the efficacy of zeolite for boron removal under controlled and real conditions was evaluated. The results indicated a notable difference in sorption behavior between the two matrices, reflecting the complexity and heterogeneity of interactions in the boron–zeolite system. In the synthetic solution, the Freundlich model provided the best fit (R^2^ = 0.9917), suggesting heterogeneous and multilayer sorption, while the Sips model showed high efficacy in describing the sorption in both matrices, evidencing its capability to capture the complex nature of the interaction between boron and zeolite under different environmental conditions. However, in natural irrigation waters, the Jovanovic model demonstrated the most accurate fit (R^2^ = 0.999), highlighting the importance of physical interactions in boron sorption. These findings underscore the significant influence of the water matrix on the efficacy of zeolite as a boron removal agent, emphasizing the need to consider the specific composition of irrigation water in the design of removal treatments. Additionally, the results stress the importance of selecting the appropriate isothermal model to predict boron sorption behavior, which is crucial for developing effective and sustainable treatment strategies. This study provides a basis for optimizing boron removal in various agricultural and industrial applications, contributing to the design of more efficient and specific water treatment processes.

## 1. Introduction

Proper water resource management in agriculture involves not only ensuring sufficient water availability but also maintaining its quality, a critical aspect in arid and semi-arid regions [1,2,3,4]. These areas, characterized by their limited availability of fresh water and high dependence on alternative sources for irrigation, face the added challenge of elevated concentrations of elements such as boron, whose fluctuations in irrigation water can seriously compromise agricultural viability [3,5,6].

Recent studies have assessed the economic viability of developing non-traditional water sources for agricultural irrigation, identifying strategies to face challenges and increase affordability by using alternative waters in agricultural sites under water stress [3]. This approach aligns with the need to adapt to the growing water demand and address costs associated with water treatment, distribution, and storage. The reuse of treated municipal wastewater, brackish water, and desalinated water have been presented as viable options to ensure agricultural production and the economic growth of the sector, despite the primary challenges represented by the high costs, energy demand, and the management of hazardous elements to soil and agriculture such as boron [3,7,8].

The toxicity of boron, even at moderate levels, can inhibit the growth of sensitive plants, reducing agricultural production and affecting local biodiversity. This situation highlights the urgent need to develop and optimize technologies for effective boron removal that are economically viable and sustainable in the long term.

Over the last decades, various studies have explored techniques to mitigate the presence of boron in waters intended for irrigation, including reverse osmosis, adsorption by ion-exchange resins, and electrodialysis techniques [9,10]. However, these technologies face significant limitations when applied on a real scale, particularly in terms of operational costs, maintenance, and efficiency against the complexity of irrigation waters that present variability in their chemical composition. In this context, research on the use of zeolites emerges as a promising avenue, given their potential to selectively adsorb boron thanks to their unique physico-chemical properties [11,12]. Nonetheless, much of the available research has predominantly focused on model systems with synthetic waters, limiting the extrapolation of results to real agricultural scenarios where the interaction between boron and other water constituents can significantly influence the removal efficiency.

This study aimed to address this research gap by focusing on the efficacy of zeolites for boron removal in real irrigation waters, directly confronting the complexity of these aqueous matrices. By investigating and comparing the adsorption dynamics under conditions that more accurately reflect actual agricultural use, we sought not only to corroborate the applicability of zeolites in practice but also to deepen the understanding of how the varied composition of irrigation waters influences the interaction between boron and the adsorbent. This approach allows us to not only validate more efficient and specific removal techniques but also to contribute to more sustainable water management in agriculture, offering viable solutions for the challenge of maintaining irrigation water quality in areas vulnerable to water scarcity and boron accumulation.

The significance of this work lies in its contribution to the development of irrigation water management strategies that are essential for food security and sustainable development in arid and semi-arid regions. By providing a solid and applicable scientific basis for boron removal, this study not only aims to improve agricultural practices in these regions but also offers a framework for future research in the optimization of water treatment technologies tailored to the specific challenges of agricultural irrigation.

## 2. Results and Discussion

The study and determination of the most suitable isothermal model to describe boron removal using zeolites are of great importance from both environmental and technological perspectives. Environmentally, boron, although an essential micronutrient, can be toxic to plants and aquatic organisms in high concentrations and poses a risk to human health [5,13,14]. Zeolites, being minerals with a porous structure and a high ion exchange capacity, have emerged as promising candidates for the removal of boron from wastewater and the mitigation of its impact on the environment [12,15].

Determining the correct isothermal model provides a deep understanding of the interactions between boron and zeolite, thus allowing the optimization of the removal process. An accurate isothermal model can predict the adsorption capacity of zeolite, inform about the affinity between the adsorbent and the adsorbate, and enable the effective design of treatment systems at an industrial scale. Furthermore, the selection of the appropriate model is crucial for the development of economical and sustainable water treatment strategies, ensuring that environmental regulations are met and public health is protected.

**Figure 1:**
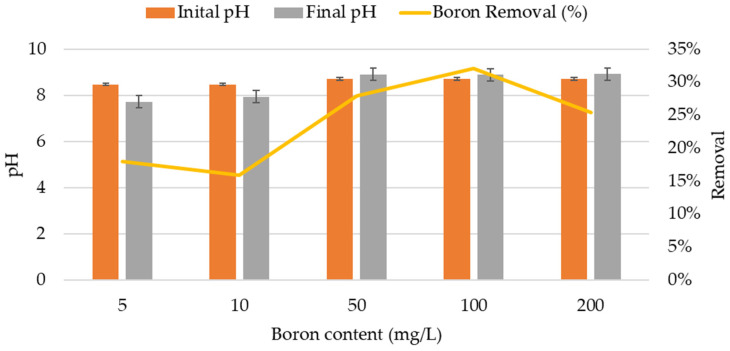
Variation in the pH of the synthetic H_3_BO_3_·H_2_O solutions and percentages of boron removal in zeolite obtained in the isothermal assays.

In this study, two different but complementary isothermal assays were conducted. In the first assay, and in accordance with the methodology traditionally used for isothermal studies, it was performed with a synthetic boron solution in order to study the specific adsorption, avoiding competition for the adsorbent’s active sites by other species. In all tests, a slight variation in pH was observed but with different trends. While in the synthetic solutions with 5 and 10 mg/L of boron, the pH decreased by between 9% and 6%, respectively; in the samples with higher concentrations (50, 100, and 200 mg/L), the pH increased by about 2–3%. The highest final pH (8.92) was determined for the synthetic samples with 200 mg/L of boron. The maximum boron removal (32%) was observed in the samples with a concentration of 100 mg/L, while the lowest removal percentages (16%) were recorded in the samples with 10 mg/L of boron (Figure 1). Although performed in resins, Sarri et al. [16] also identified the maximum boron sorption capacity at a pH equal to 9.

**Figure 2:**
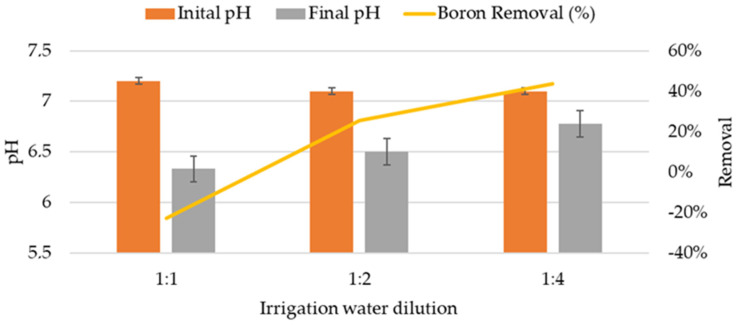
pH variation in dilutions of irrigation water samples and boron removal percentages by zeolite in isothermal assays.

The second isothermal assay was conducted with natural irrigation water with the aim of identifying the real sorption behavior with the coexistence of other chemical elements. In this assay, unlike with the synthetic samples, all tests showed a decrease in pH after 24 h of contact with the zeolite (Figure 2). Thus, while the initial pH of the irrigation water was close to neutrality (pH = 7), after 24 h, the pH decreased by between 5% and 12%. The undiluted samples showed the greatest decrease (pH = 6.33). This drop in pH is indicative of an acidification process, probably attributable to the adsorption of alkaline cations by the zeolite, which aligns with the principles of cation exchange and the known properties of zeolite as an exchange agent. Moreover, this change in pH can have direct implications on nutrient availability and the mobility of heavy metals in irrigation solutions, potentially affecting soil and plant health [12].

However, the results obtained for boron removal did not reflect a uniform trend, with removal percentages varying from −37% to 50%. This behavior suggests a complex dynamic in the interaction between boron and zeolite, where, in some cases, an increase in boron concentration in the treated water was observed. This phenomenon could be attributed to the release of previously adsorbed boron in the zeolite or competition between boron and other cations for the available adsorption sites [15]. Regarding sodium removal, the values ranged from an increase of 116% to a reduction of 73%. These results suggest that, under certain conditions, zeolite can release sodium into the water, possibly due to saturation of the exchange sites with other cations present in the aqueous matrix [17]. This behavior highlights the importance of considering the initial composition of the zeolite and its exposure history to different solutes.

Regarding calcium and potassium, the results showed a wide variation in removal percentages, with values indicating both adsorption and release of these ions. Increases in concentration of up to 164% for calcium and 468% for potassium were reported, indicative of a net desorption of these cations from the zeolite into the aqueous medium. This finding is of particular interest as it demonstrates that zeolite can act as a source of these nutrients under certain conditions, which could have applications in plant nutrition [11,18].

### 2.1. Isothermal Assays with Synthetic Water

After obtaining the experimental results, the different kinetic models considered for the removal of boron via zeolite in the samples of synthetic solutions were applied (Table 1). The isotherm graphs for the boron sorption on zeolites in synthetic water are presented in Figure S1 of the Supplementary Materials.

Considering that the application of isothermal models is essential to elucidate the interaction mechanisms between boron and zeolite as they provide a quantitative framework to interpret the sorption kinetics and capacity under controlled experimental conditions, the Langmuir model, which presupposes uniform sorption on a monolayer without interactions among adsorbed molecules, resulted in a maximum sorption capacity (*q_max_*) of 4.3626 mg/g. However, the obtained determination coefficient indicated a poor fit of this model to the experimental data, suggesting that the Langmuir isotherm is not the most appropriate description for the boron sorption process in this case, aligning with the literature for other sorbents such as clay [19]. However, Öztürk and Kavak [20] reported a good correlation with the Langmuir model for initial boron concentrations of 600 mg/L and experimental temperatures of 35 and 45 °C.

In contrast to Langmuir, the Freundlich model, which assumes a heterogeneous surface with sorption sites of variable energies, fit the experimentally obtained data with an R^2^ = 0.9917. This fit suggests that boron sorption on zeolite is characterized by a heterogeneous process, which is consistent with the physicochemical nature of the interactions between boron ions and active sites in the zeolite. The model parameters, *K_F_* and *n*, reflect a moderate sorption capacity and a physically favorable sorption process. Similar results were identified for boron removal in zeolitic imidazolate frameworks [21].

The Sips and Toth models, which attempt to combine aspects of the Langmuir and Freundlich models to describe systems with surface heterogeneity and saturation at high concentrations, also showed significant fits (R^2^ = 0.9643), which could indicate their relevance in describing boron sorption on zeolite. These models suggest that sorption is not limited to a monolayer and that the heterogeneity of sorption sites plays a significant role in the process. Similar results were obtained by Afolabi et al. [22] and Diana et al. [23] for boron sorption in fertile soils and fibrous polymeric materials, respectively.

The Jovanovic model, focused on sorption within micropores, and the Temkin model, which considers adsorbate–adsorbent interactions in sorption energy, provided reasonable fits, with R^2^ values of 0.9544 and 0.833, respectively. These results suggest the contribution of specific sorption mechanisms, such as microporosity and energetic interactions between boron and zeolite [24].

In contrast, the Dubinin–Radushkevich and Redlich–Peterson models did not adequately fit the experimental data, as indicated by the low determination coefficients and non-physical parameters obtained (*a_RP_* and *b_RP_*). This highlights the limitations of these models in describing the complexity of boron sorption on zeolite under the studied conditions.

Based on the detailed analysis of the isothermal sorption models, it was identified that the Freundlich model provides the best representation of the boron sorption process on zeolite, highlighting the heterogeneity of the sorption surface and the favorable nature of the process. Corroboration through the Sips and Toth models reinforces the understanding that sorption dynamics are complex and not limited to simple monolayer formation mechanisms or homogeneous interactions.

The choice of the Freundlich model aligns with the scientific literature, where natural zeolites tend to show adsorption behaviors that fit this model due to their heterogeneity and the complexity of solute–adsorbent interactions [25,26]. Additionally, the zeolite’s ability to adsorb boron across a wide range of concentrations indicates its potential for the remediation of boron-contaminated waters, an environmental challenge of relevance in various industries [9,12,27].

### 2.2. Isothermal Assays with Irrigation Water

For the evaluation of the boron sorption equilibrium in irrigation waters with natural zeolite, the same eight isothermal models that were applied to the synthetic solutions were analyzed, with the aim of adequately describing the sorption process, but also to identify the different behaviors of both boron and zeolite in the presence/coexistence with other chemical species. The results of the studied models are shown in Table 2, and the isotherm graphs for boron sorption on zeolites in natural irrigation water are presented in Figure S2 of the Supplementary Materials.

In more detail, the Langmuir model, which assumes sorption on a monolayer with homogeneous adsorption sites, presented a determination coefficient (R^2^) of 0.5814, indicating a moderate fit with a maximum sorption capacity of 0.288 mg/g and a Langmuir constant of 2.725 L/mg. Although this model is widely used to describe sorption on homogeneous surfaces, the moderate fit suggests that boron sorption on natural zeolite might not fully conform to this model.

On the other hand, the Freundlich model, applicable to heterogeneous surfaces, did not provide a reliable R^2^ value in this study, preventing a direct assessment of its applicability. However, more complex models like Sips, which combines features of both Langmuir and Freundlich models to describe systems with sorption heterogeneity, showed an excellent fit with an R^2^ of 0.985. This indicates that the Sips model may be particularly representative for this system, with the parameters reflecting a sorption capacity of 0.676 mg/g, a Sips constant of 4.392 L/mg, and an exponent of 1.512, indicating a strong affinity of boron for the zeolite, with behavior transitioning between monolayer and multilayer sorption depending on the boron concentration [28].

The result of the Toth model, with an R^2^ of 0.998, suggests an almost perfect fit and reflects the model’s ability to accommodate the heterogeneity of adsorption sites, providing a sorption capacity of 0.743 mg/g. This model, like the Sips model, offers a detailed understanding of the variable nature of sorption in zeolites, fitting well to systems where sorption decreases with an increase in adsorbate concentration.

The Jovanovic model, with the highest R^2^ of 0.999, indicated a predominant physical interaction in boron sorption, suggesting that Van der Waals forces play a significant role. The high sorption capacity of 0.805 mg/g underscores the effectiveness of zeolite in capturing boron, possibly due to the formation of weak but significant bonds in terms of the amount of boron removed.

Additionally, the Temkin and Dubinin–Radushkevich models, despite their moderate fits, provide valuable insights into the sorption energies and the potential existence of porosity and microporosity in the zeolite, respectively. These models suggest that both chemical interactions and the physical characteristics of the zeolite influence boron sorption.

Finally, the Redlich–Peterson model, with an R^2^ of 0.7094, failed to effectively capture the sorption behavior of the studied system. This could be due to the complexity of the sorption system requiring a more detailed description than that provided by this model.

### 2.3. Evaluation of Zeolite Behavior

The comparative analysis of boron sorption equilibrium assays using zeolite in a synthetic solution containing only boron versus natural irrigation waters revealed critical differences in efficiency and the underlying sorption mechanism, underscoring the significant influence of the water matrix on the sorption process. These differences not only reflect variability in the boron–zeolite interactions but also have important implications for the practical application of zeolite in boron removal treatments.

The results of the Langmuir model, which exhibited a poor fit in the synthetic solution (R^2^ = 0.0725) and a moderate fit in natural irrigation waters (R^2^ = 0.5814), highlight the inadequacy of this model to capture the complexity of boron sorption in the presence of other components. The monolayer sorption assumed by the Langmuir model is challenged by the presence of multiple species in irrigation water, which can interfere or compete with boron for sorption sites, or even alter the surface structure of the zeolite, affecting its sorption capacity.

Similarly, the high efficacy of the Freundlich model for describing sorption in the synthetic solution (R^2^ = 0.9917) contrasts with its inapplicability in natural irrigation waters, suggesting that surface heterogeneity and multilayer sorption are more representative under controlled conditions. However, the complexity and presence of various substances in irrigation waters may require more sophisticated models that consider more complex interactions.

In Figure 2, an intriguing pattern can be noted where higher dilution ratios in the irrigation water correspond with increased boron removal percentages. This phenomenon can be correlated with the Freundlich adsorption isotherm, which provided the best fit for our data, as shown in Table 5. The Freundlich model is well-known for describing adsorption processes where the adsorption surface is heterogeneous, which is typically the case with natural zeolite used in varying water compositions. The enhanced boron removal at higher dilutions may be attributed to the reduced competition for active adsorption sites on the zeolite. As the concentration of competing ions decreases, more sites become available for boron, thus increasing its removal efficiency. This scenario is perfectly captured by the Freundlich model, which assumes that the adsorption capacity is related to the concentration of the adsorbate in the solution at non-uniform adsorption sites. The mathematical expression of the Freundlich model, *qe* = *K_F_C_e_*^1/*n*^, where *K_F_* and *n* are constants indicative of adsorption capacity and intensity, respectively, supports the observation that with decreasing boron concentration due to dilution, the adsorption capacity increases, showing a non-linear relationship that is characteristic of heterogeneous surfaces. This correlation is significant as it not only supports the suitability of the Freundlich model for our system but also underscores the importance of considering the effect of the solution matrix and competing ions in practical applications of zeolite for water treatment.

The Sips and Toth models offered similar and elevated fits for both the synthetic solution and irrigation waters, highlighting their ability to integrate features of monolayer and multilayer sorption, as well as the surface heterogeneity of zeolite. The results of these models, with their high determination coefficients (R^2^ close to 0.9643 and higher for both cases), indicate they can effectively capture the complexity of boron sorption across different water matrices. The similarity in the fit of these models suggests that, regardless of the matrix, certain fundamental characteristics of boron sorption remain constant, such as the existence of a limited sorption capacity and the importance of surface heterogeneity.

The results of the Jovanovic model, with the best fit for natural irrigation waters (R^2^ = 0.999), highlight the relevance of considering physical interactions, like Van der Waals forces, in more complex environments. This suggests that boron sorption may be significantly influenced by physical mechanisms under real conditions, an important consideration for treatment system design.

In this study, significant attention was given to the role of pH in altering the speciation of boron in both synthetic solutions and natural irrigation water. As known from the literature, boron predominantly exists as boric acid (B(OH)_3_) in acidic environments and as borate (B(OH)^−^_4_) in alkaline conditions [9,10]. Our experimental results confirmed that pH variations were evident, showing a decrease in synthetic solutions and a more pronounced decrease in irrigation water over the duration of the assays. These pH changes are expected to influence the speciation of boron, thereby affecting its interaction with the zeolite used. For example, negatively charged borate may have different interaction dynamics with the zeolite’s cation exchange sites compared to neutral boric acid, potentially affecting the adsorption capacity and the efficiency of the sorption process. The differential interaction based on boron species and pH might also explain the varying performance of the isothermal models applied in this study. In the synthetic solution where the pH was controlled and less variable, models predicting monolayer adsorption like the Langmuir model did not fit the data well, suggesting more complex interactions at the surface. Conversely, in natural irrigation waters with fluctuating pH levels, models that accommodate more complex and heterogeneous surface interactions such as the Jovanovic and Sips models showed a better fit, likely reflecting the variable nature of boron speciation under different pH conditions.

It is pertinent to note that the existing scientific literature on boron removal using zeolites has primarily been conducted under controlled conditions using synthetic waters. These studies have provided valuable insights into the sorption mechanisms and the capacity of zeolites to capture boron under ideal conditions, where the presence of other solutes is minimal or carefully controlled. However, applying these findings to the treatment of agricultural irrigation waters presents significant challenges, as these waters have a much more complex and variable chemical matrix. The presence of various solutes in agricultural waters, including organic matter, dissolved salts, and other competing ions, can notably influence the efficiency of zeolite for boron removal, altering both the capacity and kinetics of sorption. This study sought to address this research gap by providing critical data on the effectiveness of zeolites in removing boron from real agricultural irrigation waters. Through comparing our results with those obtained in synthetic aqueous systems, it is evident that the complex matrix of agricultural waters can significantly modify the sorption behavior observed in previous studies, highlighting the importance of conducting research in real application contexts for a comprehensive and practical understanding of water treatment technologies.

## 3. Materials and Methods

### 3.1. Sorbent Material: Zeolite

For this study, commercial natural zeolite of the clinoptilolite type FERTCEL (Zeocel Ltda., Águeda, Portugal), commonly used in agriculture to regulate soil fertility, maximize nutrient utilization, and both qualitatively and quantitatively improve harvests, was used. The physicochemical characterization of the zeolite, which was conducted and published in a previous work, is shown in Table 3 [29]. Prior to its incorporation into the experiments, the zeolite was subjected to a pre-wash with distilled water to remove any surface residue such as dust from the granules, without any additional chemical treatment. This step is crucial to ensure that the original characteristics of the material are not altered, thus allowing for a reliable study of its sorption properties as specified by the manufacturer. It is noteworthy that this work is based on, and therefore is a continuation of, the kinetic studies that were already conducted on the subject [29].

### 3.2. Batch Isothermal Tests

The sorption efficacy was evaluated through batch equilibrium assays, following an experimental design that contemplates variations in the initial boron concentration, but with a constant zeolite content and contact time. The adsorption equilibrium experiments were carried out by varying the initial boron concentrations (5, 10, 50, 100, and 200 mg/L) in synthetic solutions prepared with H_3_BO_3_·H_2_O (PanReac, AppliedChem, Darmstadt, Germany). For all assays, 100 mL of the solutions was placed in non-sterile polypropylene Erlenmeyer flasks with a total capacity of 250 mL and 4.93 g/L of zeolite was added. Subsequently, the flasks were covered with plastic film to prevent the entry of environmental dirt. The solutions with the zeolite were maintained for 24 h at a constant temperature (23 ± 2 °C). At the end of the contact time, the mixture was quickly filtered through 0.45 μm cellulose acetate filters. The amount of zeolite to be used was experimentally defined as a result of a prior study of factorial experimental planning and its sorption kinetics [29]. Once the samples were filtered, the boron content was determined using a boron chemical test through titration with boric acid (HI38074 method, Hanna Instruments, Woonsocket, RI, USA). A parallel control experiment without zeolite was also carried out to identify and discard possible intrinsic variations in the synthetic samples. The pH of the solutions was controlled before and after the assay with the zeolite. In all cases, the assays were conducted in triplicate (*n* = 3) and the results are shown as the average values obtained.

The use of boron concentrations ranging from 5 to 200 mg/L in synthetic solutions is based on the need to fully understand the adsorption properties of zeolites in various practical scenarios. This strategy ensures that the findings are relevant for agricultural and environmental applications where boron contamination can be higher than in typical natural water sources. Using high concentrations, such as 200 mg/L, allows us to simulate extreme conditions that might be encountered in certain industrial environments or in reclaimed waters, such as treated municipal wastewater used for agricultural irrigation in arid regions. These waters can contain high levels of boron due to the inefficiency of conventional treatments. Additionally, the accumulation of boron in soil and its phytotoxic effects on crops highlight the importance of understanding the adsorption behavior of zeolites at different concentrations. This knowledge is vital to ensuring the technology’s effectiveness in situations where boron levels exceed typical environmental concentrations, thus protecting crop health and productivity. Evaluating adsorption at both low and high concentrations allows for a comprehensive characterization of the adsorption isotherm of zeolites, providing a detailed understanding of the maximum adsorption capacity and the efficiency of boron removal. The comparability of results across different concentrations is scientifically valid and provides critical information on the efficiency and applicability of zeolites in diverse real-world conditions. By establishing the relationship between concentration and adsorption capacity, we can better predict the behavior of the adsorbent in different water matrices, ensuring that the findings are applicable to a wide range of practical scenarios and can significantly contribute to the field of water resource management, especially in regions with water scarcity and high levels of boron.

Furthermore, equilibrium assays with natural irrigation water were conducted in order to verify the interferences/synergies with other chemical species [30]. For this, irrigation water samples were collected from a pond located on a commercial farm situated in Cuevas del Almanzora (Almería, Spain). The boron concentrations were determined following the same methodology as for the synthetic solutions, and the concentration of other chemical species of agronomic interest, such as Ca, Na, and K, were determined using an Imacimus Multi Ion 10 portable multi-ion nutrient analyzer (Imacimus NT Sensors, El Catllar, Spain).

Different dilutions of irrigation water (1:1, 1:2, and 1:4 *v*/*v*) were used for the sorption equilibrium experiments. The initial characterization of the irrigation water sample dilutions used in this study is shown in Table 4. The experimental conditions (zeolite content, contact time, and temperature) were the same as for the synthetic solutions.

### 3.3. Isothermal Models

For the interpretation of equilibrium data, eight theoretical sorption models were applied, including Langmuir, Freundlich, Sips, Toth, Jovanovic, Temkin, Dubinin–Radushkevich (D-R), and Redlich–Peterson [22,28,31,32,33,34,35,36]. The specific equations for each of the studied models are presented in Table 5. The selection of these models allowed for a comprehensive understanding of the adsorption process, ranging from monolayer formation to surface heterogeneity and adsorption energetics.

The Langmuir model is based on the assumption that adsorption occurs at specific sites on the adsorbent’s surface, forming a monolayer, without interactions between the adsorbed molecules at adjacent sites, with a maximum adsorption capacity (*q_max_*) and an adsorption constant (*K_L_*) that describes the affinity between the adsorbent and the adsorbate [32]. Unlike Langmuir, the Freundlich model applies to heterogeneous surfaces, describing adsorption that is not limited to the formation of monolayers and is characterized by constants that indicate the capacity and intensity of adsorption (*K_F_*, and *n*, respectively) [35].

On the other hand, the Sips model combines the properties of the Langmuir and Freundlich models, offering a more accurate description over the entire range of adsorbate concentrations. It reduces to the Langmuir model at low concentrations and to the Freundlich model at high concentrations [28]. Meanwhile, the Toth model introduces modifications to account for the adsorbent surface’s heterogeneity. This is particularly useful for describing adsorption in systems where the isotherm shows a pronounced curvature at low concentration [36]. The Jovanovic model assumes that adsorption occurs in micropores and is similar to the Langmuir model but considers the existence of interactions between the adsorbed molecules and the adsorbent. Its equation resembles that of Langmuir with a corrective term for low concentrations [34]. Conversely, the Temkin model addresses adsorbate–adsorbent interactions and their impact on adsorption energy, assuming that it decreases linearly with surface coverage [33]. The Dubinin–Radushkevich model is distinguished by differentiating between physical and chemical adsorption, using Gibbs free energy as the basis for its formulation [37]. Finally, the Redlich–Peterson model provides a versatile framework that integrates features of the Langmuir and Freundlich models, which is applicable to heterogeneous systems without being limited to specific monolayer or multilayer descriptions [36].

The equations of the models were fitted to the experimental data using regression with the STATISTICA 18 v. 13.3.0 StatSoft software (TIBCO Software Inc., Palo Alto, CA, USA). The parameters of interest for each of the studied models were calculated from the model fits.

## 4. Conclusions

This study provides a comprehensive comparative assessment of boron sorption dynamics using zeolites in irrigation water solutions through the application of various isothermal models. The results highlight the efficacy of zeolites in removing boron, showing significant differences in sorption behavior between synthetic solutions and natural irrigation waters. This variability underscores the influence of the aqueous matrix on the boron–zeolite interactions and highlights the importance of selecting the appropriate isothermal model to accurately predict the sorption capacity.

In the case of synthetic solutions, the Freundlich model provided the best fit, indicating heterogeneous and multilayer sorption. This result suggests that the porous structure and surface characteristics of the zeolite facilitate the adhesion of boron ions at different energy sites, which is characteristic of systems with moderate interaction complexity and without the presence of significant competitors. Specifically, the zeolite demonstrated a maximum sorption capacity of 12.9776 mg/g under these conditions, a captivating figure that demonstrates the potential of zeolite in scenarios where the boron concentration varies.

On the other hand, the Jovanovic model stood out in describing boron sorption in natural irrigation waters, particularly at higher boron concentrations (50 to 200 mg/L). This model emphasizes the importance of physical interactions and suggests that despite the presence of other solutes and competition for sorption sites, the zeolite’s efficacy in capturing boron is not significantly reduced. The observed maximum sorption capacity in this matrix was notably high at 0.805 mg/g, highlighting the zeolite’s robust ability to transcend environmental variations and effectively remove boron. This finding is particularly relevant for designing water treatment systems in agriculture, where the water quality can be highly variable, and the efficiency in removing contaminants must be maintained despite the presence of multiple chemical species.

The variability in model performance between the two water matrices underlines the need for the careful evaluation of the specific water conditions when applying zeolite for boron removal. The results imply that while certain isothermal models can adequately describe boron sorption under ideal or simplified conditions, the presence of other substances in natural irrigation water can significantly alter the sorption dynamics. This has critical practical implications, suggesting that zeolite-based boron removal treatments must be carefully calibrated and possibly customized to the specific characteristics of the irrigation water in use. Additionally, these findings emphasize the importance of conducting sorption tests under conditions that, as closely as possible, reflect the intended end-use, ensuring the efficacy and reliability of boron removal treatments in agricultural and industrial applications.

Future research should focus on evaluating the regeneration and reuse of zeolites used for boron removal, as well as exploring the impact of the presence of other ions on the sorption efficacy. This approach will not only enable the design of more effective and sustainable water treatment systems but also facilitate the development of agricultural practices resilient to irrigation water scarcity and changes in quality.

## Figures and Tables

**Table 1:** Results of the application of isothermal models for boron removal in synthetic water: equations, determination coefficients (R^2^), and model parameters.

Isothermal Model	Linearized Equation	R^2^	Model Parameters *
Langmuir	*y =* 0.01*x* + 3.78	0.0725	*q_max_* = 4.3626 *K_L_* = −4.1572 × 10^6^
Freundlich	*y* = 0.07*x* + 0.61	0.9917	*K_F_* = 0.1955 *n* = 1.2616
Sips	*y* = 0.07*x* + 0.30	0.9643	*q_s_* = 12.9776 *K_s_* = 6.6144 × 10^−4^ *n_s_* = 1.7162
Toth	*y* = 0.06*x* + 0.33	0.96	*q_T_* = 6.10 *K_T_* = 40.89 *t* = 0.80
Jovanovic	*y* = 0.06*x* + 1.28	0.9544	*q_J_* = 1.9525 *K_J_* = −1.1109 × 10^−2^
Temkin	*y* = 2.42*x* − 3.66	0.833	*A*: 0.220 *B*: 2.418
Dubinin–Radushkevich	*y* = 0.00*x* + 0.01	0.525	*q_DR_*: 26.38 *K_DR_*: 0.519
Redlich–Peterson	-	0.888	*K_RP_*: 0.000030 *a_RP_*: −0.9999 *b_RP_*: −0.000058

* See Table 5 for the definition of symbols.

**Table 2:** Results of the application of isothermal models for boron removal in natural irrigation water: equations, determination coefficients (R^2^), and model parameters.

Isothermal Model	Linearized Equation	R^2^	Model Parameters *
Langmuir	y=26.73x−9.1383	0.5814	*q_max_* = 0.288 *K_L_* = 2.725
Freundlich	$y=0.92291+\frac{1}{44711}x$	−8.45 × 10^−9^	-
Sips	y=0.0652x+0.7911	0.985	*q_s_* = 0.676 *K_s_* = 4.392 *n_s_* = 1.512
Toth	y=0.667x+0.000	0.998	*q_T_* = 0.743 *K_T_* = 1.764 *t* = 0.705
Jovanovic	y=0.547x+0.942	0.999	*q_J_* = 0.805 *K_J_* = 0.294 *n* = 0.737
Temkin	y=−1.4208x+2.2717	0.6722	*A*: −1.4208 *B*: 2.2717
Dubinin–Radushkevich	$y=-2.7621x-5.031\times 10^{-6}$	0.6381	*q_DR_*: −2.76216 *K_DR_*: 5.031 × 10^−6^
Redlich–Peterson	-	0.7094	*K_RP_*: 5.3766 × 10^5^ *a_RP_*: 1.7039 × 10^4^ *b_RP_*: 4.361

* See Table 5 for the definition of symbols.

**Table 3:** Average chemical composition (%) and physical characteristics of the used natural zeolite.

Chemical Composition (%)		Physical Characteristics		
SiO_2_	63.00	Surface area	m^2^/g	40
Al_2_O_3_	11.57	Bulk density	g/L	0.98
CaO	5.78	Cation exchange capacity	mg/g	1.8
Na_2_O	2.39	Particle size	mm	1.0–2.5
Fe_2_O_3_	1.82			
FeO	0.81			
K_2_O	1.49			
TiO_2_	0.45			
MgO	0.92			
P_2_O_5_	0.09			
pH	8.2			

**Table 4:** Initial characterization of the irrigation water used.

	B	Ca	Cl	K	Na	NH_4_	NO_3_	pH
mg/L	6.4	85.75	833.25	44	232	1.44	450.25	7.2

**Table 5:** Sorption models used for the study of boron removal in zeolite and their corresponding equations and parameters.

Model	Equation	Parameters
Langmuir	$q_{e}=\frac{q_{max}K_{L}C_{e}}{1+K_{L}C_{e}}$	*q_e_* is the amount of adsorbate per unit mass of adsorbent at equilibrium (mg/g), *C_e_* is the adsorbate concentration in the solution at equilibrium (mg/L), *q_ma_*_x_ is the maximum adsorption capacity representing the formation of a complete monolayer (mg/g), and *K_L_* is the Langmuir adsorption constant (L/mg).
Freundlich	$q_{e}=K_{F}C_{e}^{\frac{1}{2}}$	*K_F_* (mg ^(1−1/*n*)^ L^(1/*n*)^/g) and *n* are constants that indicate the adsorption capacity and intensity, respectively. A value of *n* > 1 suggests favorable adsorption.
Sips	$q_{e}=\frac{q_{max}K_{S}C_{e}^{nSips}}{1+K_{S}C_{e}^{nSips}}$	*K_S_* is a constant related to adsorption affinity and *n_Sips_* is a parameter that indicates surface heterogeneity.
Toth	$q_{e}=\frac{q_{max}K_{T}C_{e}}{{\left(1+{\left(K_{T}C_{e}\right)}^{t}\right)}^{1/t}}$	*K_T_* is the Toth constant related to adsorption energy and *t* is a parameter that describes the surface heterogeneity.
Temkin	$q_{e}=\frac{RT}{b_{T}}\ln\left(K_{T}C_{e}\right)$	*R* is the universal gas constant, *T* is the temperature (*K*), *b_T_* (also labeled as B) is the Temkin constant related to the heat of adsorption per molecule of adsorbate as the layer is completed (J/mol), and *K_T_* (also labeled as A) is an equilibrium constant corresponding to the maximum binding energy (L/g).
Dubinin–Radushkevich	$q_{e}=q_{max}exp\left(-\beta \varepsilon^{2}\right)$	*ε* is Polanyi’s energy, *β* is a constant related to free adsorption energy, and *q_max_* is the maximum adsorption capacity.
Redlich–Peterson	$q_{e}=\frac{K_{RP}C_{e}}{1+\alpha_{RP}C_{e}^{b_{RP}}}$	*K_RP_*, *a_RP_*, and *b_RP_* are model constants. This model is not limited to a specific monolayer or multilayer description, making it versatile for a wide range of conditions.
Jovanovic	$q_{e}=q_{max}exp\left(-K_{J}C_{e}\right)$	*K_J_* is the Jovanovic constant.

## Data Availability

The data presented in this study are available in the present work.

## Supplementary Materials

The following supporting information can be downloaded at: https://www.mdpi.com/article/10.3390/molecules29112545/s1, Figure S1. Graphical representation of Langmuir, Freundlich, Sips, Toth, Jovanovic, Temkin, Dubinin-Radushkevich, and Redlich-Peterson isotherm models used to study boron sorption by zeolite in synthetic water. Figure S2. Graphical representation of Langmuir, Freundlich, Sips, Toth, Jovanovic, Temkin, Dubinin-Radushkevich, and Redlich-Peterson isotherm models used to study boron sorption by zeolite in natural irrigation water.
