# Supplementary material for: Comparative Evaluation of Boron Sorption Dynamics on Zeolites in Irrigation Waters: An Isothermal Modeling Approach
Source: Molecules. 2024 May 28;29(11):2545. doi: 10.3390/molecules29112545 (PMC11173699; doi:10.3390/molecules29112545)

Comparative Evaluation of Boron Sorption Dynamics on Zeolites in Irrigation Waters: An Isothermal Modeling Approach

Dámaris Núñez-Gómez, Juan José Martínez-Nicolás, Pilar Legua *, Carlos Giménez-Valero, Alejandro Andy Maciá-Vázquez and Pablo Melgarejo
Plant Production and Microbiology Department, Miguel Hernandez University (UMH), Ctra. Beniel Km 3.2, 03312 Orihuela, Alicante, Spain.
* Correspondence: p.legua@umh.es

SUPPLEMENTARY MATERIALS

Figure S1. Graphical representation of Langmuir, Freundlich, Sips, Toth, Jovanovic, Temkin, Dubinin-Radushkevich, and Redlich-Peterson isotherm models used to study boron sorption by zeolite in synthetic water.

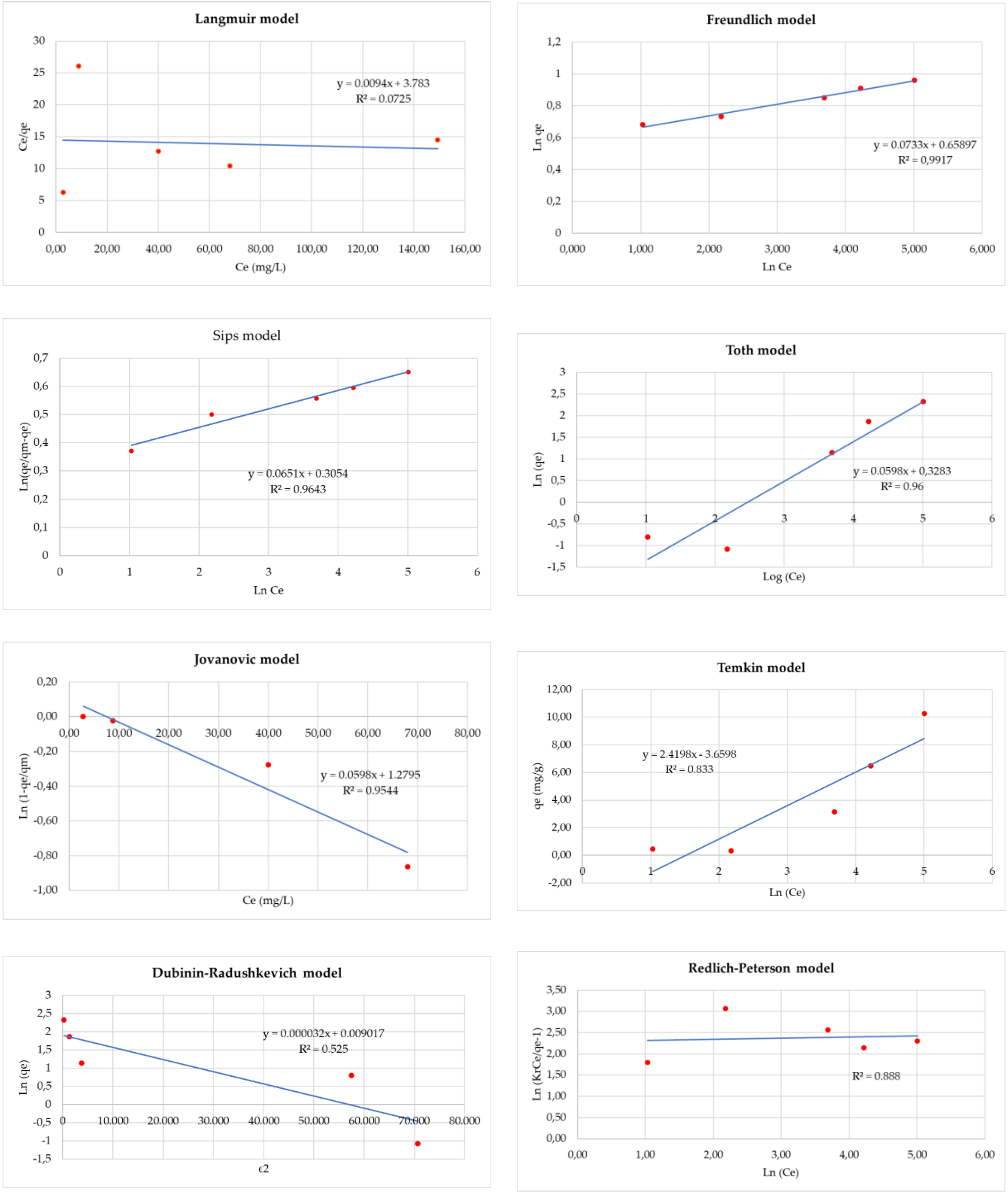

Figure S2. Graphical representation of Langmuir, Freundlich, Sips, Toth, Jovanovic, Temkin, Dubinin-Radushkevich, and Redlich-Peterson isotherm models used to study boron sorption by zeolite in natural irrigation water.

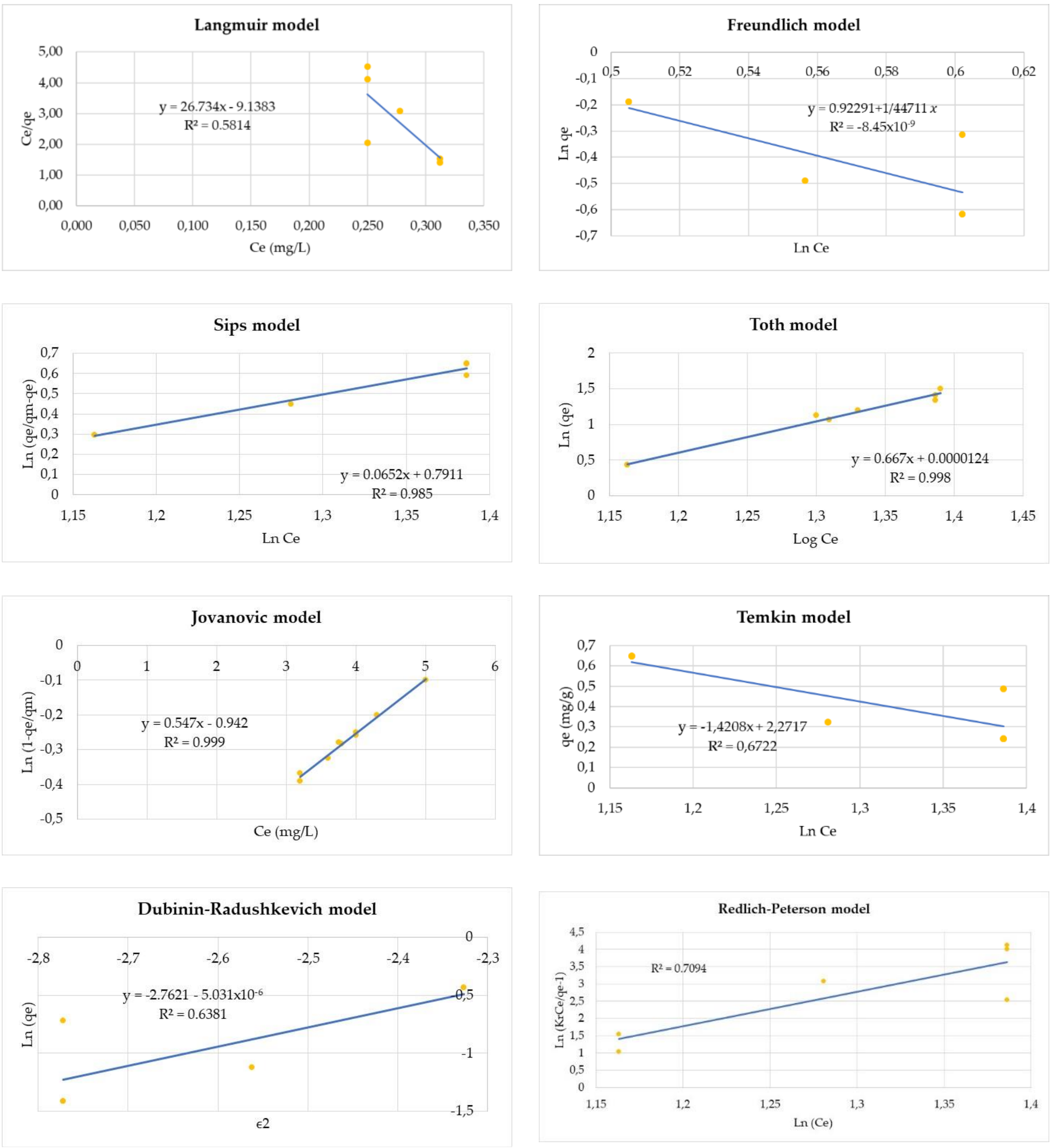

Supplement: Supplementary file 1 [file molecules-29-02545-s001.zip › molecules-2982419-supplementary.pdf]
